# Assessment of Model Based (Input) Impedance, Pulse Wave Velocity, and Wave Reflection in the Asklepios Cohort

**DOI:** 10.1371/journal.pone.0141656

**Published:** 2015-10-29

**Authors:** Bernhard Hametner, Stephanie Parragh, Christopher Mayer, Thomas Weber, Luc Van Bortel, Marc De Buyzere, Patrick Segers, Ernst Rietzschel, Siegfried Wassertheurer

**Affiliations:** 1 Health & Environment Department, AIT Austrian Institute of Technology, Vienna, Austria; 2 Department of Analysis & Scientific Computing, Vienna University of Technology, Vienna, Austria; 3 Cardiology Department, Klinikum Wels-Grieskirchen, Wels, Austria; 4 Department of Pharmacology, Ghent University, Ghent, Belgium; 5 Institute of Biomedical Technology, iMinds Medical IT, Ghent University, Ghent, Belgium; 6 Department of Internal Medicine, Ghent University, Ghent, Belgium; Medical University of Graz, AUSTRIA

## Abstract

**Objectives:**

Arterial stiffness and wave reflection parameters assessed from both invasive and non-invasive pressure and flow readings are used as surrogates for ventricular and vascular load. They have been reported to predict adverse cardiovascular events, but clinical assessment is laborious and may limit widespread use. This study aims to investigate measures of arterial stiffness and central hemodynamics provided by arterial tonometry alone and in combination with aortic root flows derived by echocardiography against surrogates derived by a mathematical pressure and flow model in a healthy middle-aged cohort.

**Methods:**

Measurements of carotid artery tonometry and echocardiography were performed on 2226 ASKLEPIOS study participants and parameters of systemic hemodynamics, arterial stiffness and wave reflection based on pressure and flow were measured. In a second step, the analysis was repeated but echocardiography derived flows were substituted by flows provided by a novel mathematical model. This was followed by a quantitative method comparison.

**Results:**

All investigated parameters showed a significant association between the methods. Overall agreement was acceptable for all parameters (mean differences: -0.0102 (0.033 SD) mmHg*s/ml for characteristic impedance, 0.36 (4.21 SD) mmHg for forward pressure amplitude, 2.26 (3.51 SD) mmHg for backward pressure amplitude and 0.717 (1.25 SD) m/s for pulse wave velocity).

**Conclusion:**

The results indicate that the use of model-based surrogates in a healthy middle aged cohort is feasible and deserves further attention.

## Introduction

The consequences of arterial stiffening on aortic hemodynamics and left ventricular load are actually considered to be major determinants of cardiovascular risk beyond established risk factors [1,2]. Starting from the late 1960’s, methods were established to quantify arterial stiffness and wave reflection at the proximal aorta under different conditions based on the concept of vascular impedance [3,4]. Research was mainly driven by invasive data assessment in the early days, but the technical development of non-invasive sensor systems like Doppler ultrasound and vascular tonometry of superficial arteries facilitated non-invasive data capturing [5]. For example, carotid to femoral pulse wave velocity measurement evolved using these techniques and proofed its clinical usefulness [6,7]. Nevertheless, the acquisition of pulse waves or left ventricular outflow remains time consuming and requires skilled operators as well as dedicated devices. Subsequently, mathematical methods were investigated to substitute measured flow waveforms by approximate or model-predicted alternatives [8,9]. Based on this simplification, new research applications became feasible and parameters based on non-invasively assessed pressure alone turned out to predict cardiovascular events in different cohorts independent of established risk factors [10–12]. Such mathematical models potentially allow the calculation of surrogates of characteristic impedance (Zc) [9,13,14]. Zc is a descriptor of combined geometrical and mechanical arterial wall properties, a major determinant of pulse wave velocity, and directly linked to vascular load [15–17]. A recently introduced model-based approach, which is grounded on a modified Windkessel system, aims to allow the calculation of input as well as characteristic impedance, pulse wave velocity and wave reflections from pressure waveforms alone [18]. The aim of this work is the comparison of Windkessel derived parameters against directly measured values in an independent large cohort (from the ASKLEPIOS study [19,20]).

## Methods

### Study population

All data was selected from the ASKLEPIOS study which is a prospective longitudinal study intended to investigate the development of cardiovascular disease in the general population. The actual study population used here includes 1163 women and 1063 men within an age range from 35 to 56 years. The study was conducted on-site in Erpe Mere, Belgium, with approval of the ethics committee of the Ghent University Hospital. All subjects agreed to join the study by written informed consent. Rationale, design, methods and baseline characteristics of the ASKLEPIOS study have been extensively published elsewhere [19]. Therefore, only a very brief methods summary will be given in the following paragraph and basic characteristics in Table A in S1 File. All parameters assessed by the methods described in the ASKLEPIOS study outline will be referred to as “Asklepios” throughout the manuscript.

### Data assessment

Aortic flow waveforms were captured by Echo-Doppler measurements and stroke volume subsequently from the cross sectional area of the left ventricular outflow tract by a Vivid7 ultrasound machine (GE Vingmed Ultrasound). Carotid pulse waveform readings were performed by vascular tonometry of the left common carotid artery. Absolute pressure calibration was done by brachial mean and diastolic pressure as described in [21]. All measurements were performed consecutively by a single, well trained operator (E.R.). Input impedance was calculated following Fourier decomposition of an averaged pressure and flow waveform, and defined as the ratio of corresponding harmonics of pressure and flow. Zc was assessed in the frequency domain and calculated as the average of harmonics 3 to 10 with exclusion of outliers [20]. Carotid pulse waveforms (Pc) were separated into their forward (Pf) and backward (Pb) traveling components using wave separation analysis (WSA). The reflection magnitude (RM) is calculated as the ratio of backward and forward wave amplitudes. Systemic vascular resistance (SVR) was set equal to the 0 Hz frequency of input impedance (ratio of mean pressure and flow). Aortic stiffness was assessed by means of carotid-femoral pulse wave velocity (Asklepios cfPWV).

### ARCSolver

**Fig 1:**
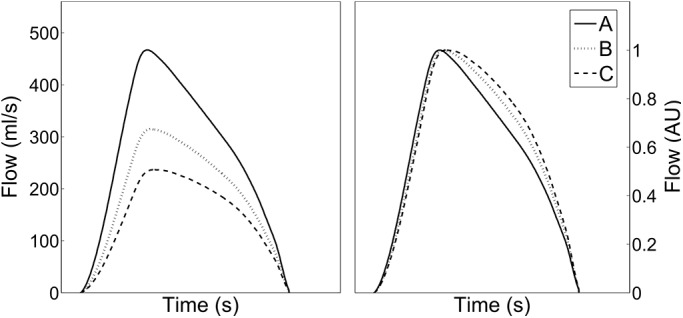
Changes in the shape of the modeled flow wave and the resulting stroke volume SV (ml) for a given heart rate HR and blood pressure level depending on arterial compliance Ca (ml/mmHg) and systemic vascular resistance SVR (mmHg*s/ml). Left: real scale, right: normalized to a height of 1 arbitrary unit (AU). Parameter values for A (Ca = 0.8, SVR = 1.0, SV = 99), B (Ca = 1.0, SVR = 1.4, SV = 71) and C (Ca = 1.2, SVR 1.8, SV = 55).

The ARCSolver (AIT Austrian Institute of Technology, Vienna, Austria) method is intended to mathematically describe left ventricular outflow during systole according to a given pressure wave. The model combines a modified 3-element like Windkessel system and transmission line theory. This approach allows the estimation of static hemodynamic parameters like stroke volume (SV) or systemic vascular resistance (SVR) as well as measures of pulsatile hemodynamics, like Pf or Pb by means of wave separation analysis and PWV as a derivative of Zc. A detailed technical description and validation in the initial cohort [9,18,22–24] as well as data on its predictive value is given elsewhere [11,25]. Fig 1 illustrates the basic principle of the method. Basically, the algorithms were designed for the use with aortic waveforms but in this study we utilized carotid waveforms as a surrogate for the first time. To be able to process carotid waveforms of the ASKLEPIOPS cohort we were obliged to slightly adapt the existing modelling approach: To identify the parameters in the Windkessel model, an optimization routine based on the minimization of left ventricular work is used [18]. Here, we modified the initial values for the parameter estimation process, but left the model, all other parameter settings and algorithms unchanged. Compared to known static flow waveform models [26], the Windkessel based ARCSolver aortic flow wave changes depending on arterial compliance and SVR, as shown in Fig 1. No additional anthropometric inputs are needed for the mathematical model for stroke volume and blood flow wave shape, although information on age and sex are used for signal pre- and post-processing as described earlier [18, 22].

### Statistics

Unless stated otherwise, results are expressed as mean and standard deviation (SD) in the statistical analysis. Data of the comparisons were analyzed using the method of Bland-Altman [27]. The correlation between variables was calculated using Pearson’s correlation coefficient. To analyze determinants of estimated and measured data, regression analysis was applied. If not stated otherwise, a level of significance of p = 0.05 was used in all tests. Analyses were performed using MedCalc 12.3 (MedCalc software, Mariakerke, Belgium).

## Results

**Fig 2:**
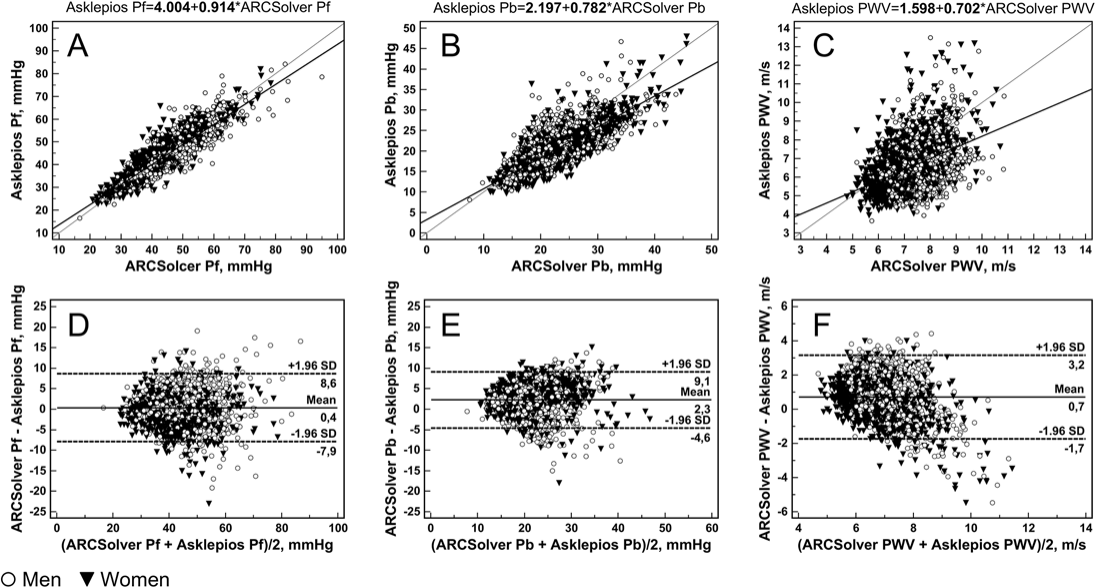
Scatter plots with regression lines and Bland Altman plots comparing the amplitudes of the forward (A, D) and backward (B, E) traveling pressure waves obtained with the ARCSolver method and the Doppler-ultrasound flow, as well as the estimated ARCSolver pulse wave velocity and the measured Asklepios carotid-femoral PWV (C, F). Bold letters in the regression equations indicate P<0.001.

**Fig 3:**
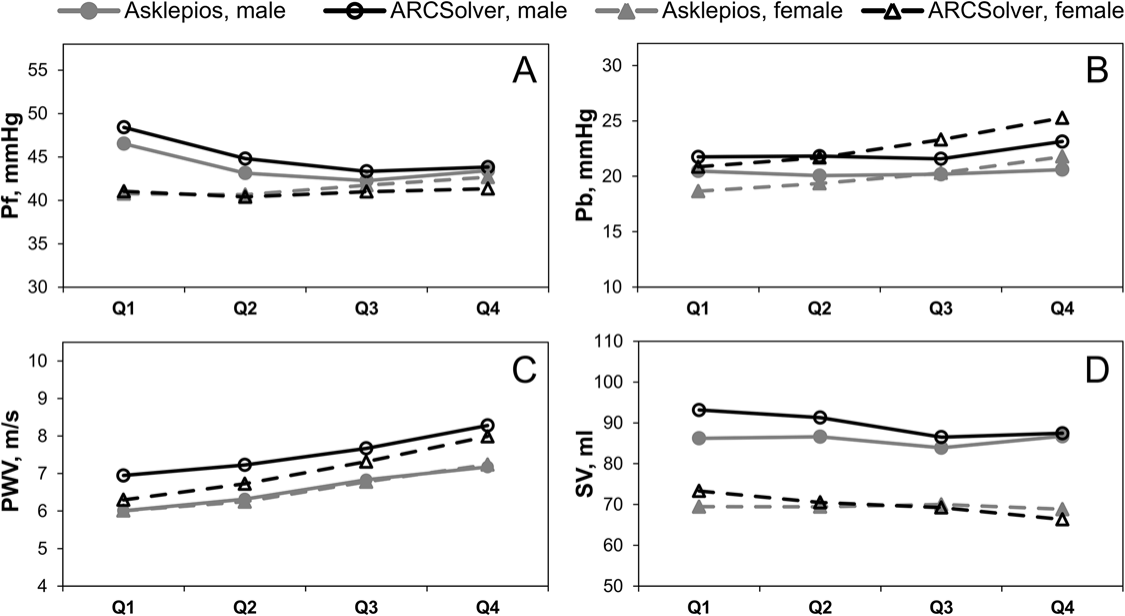
Amplitude of the forward Pf and backward Pb traveling pressure wave (A, B) and pulse wave velocity (C) as functions of sex and age. Q1, 35–40 years, 277/300 (m/w); Q2, 41–45 years, 274/303 (m/w); Q3, 46–50 years 265/281 (m/w); Q4, 51–56 years, 247/279 (m/w).

Results for the whole study population and for men and women separately are presented in Table 1. Detailed results of subgroup analysis according to age and sex are given in Table B in S1 File. All investigated parameters showed a significant association between the methods. In detail, mean amplitudes of separated forward pressure waves (Pf) are 43.0 (9.48 SD) mmHg and 42.6 (9.28 SD) mmHg for ARCSolver and Asklepios method, respectively, with a mean difference of 0.356 (4.21 SD) mmHg. Correlation is close with Pearson R = 0.90, compare Fig 2A. Mean amplitudes of the backward pressure waves (Pb) are 22.4 (5.44 SD) mmHg and 20.1 (5.22 SD) mmHg with a mean difference of 2.26 (3.51 SD) mmHg. For Pb, correlation is R = 0.78 (Fig 2B). A Bland-Altman analysis for both parameters is shown in Fig 2D and 2E. In a subgroup analysis, similar trends over age for both methods can be seen except for a slight offset in Pb, compare Fig 3A and 3B. The mean ratio or reflection magnitude (RM) of Pb and Pf is 0.529 (0.103 SD) and 0.477 (0.0872 SD) for Windkessel and Doppler based methods respectively with a significant correlation of R = 0.63.

**Table 1:** Comparison of hemodynamic parameters in the total study population and per gender group.

Parameter	Total (2226)	Men (1063)	Women (1163)
Pf, mmHg			
Asklepios	42.6 (9.28 SD)	43.9 (9.51 SD)	41.4 (8.92 SD)
ARCSolver	43.0 (9.48 SD)	45.2 (9.67 SD)	40.9 (8.84 SD)
Difference	0.356 (4.21 SD)	1.28 (4.41 SD)	-0.492 (3.84 SD)
Pb, mmHg			
Asklepios	20.1 (5.22 SD)	20.3 (5.11 SD)	20.0 (5.32 SD)
ARCSolver	22.4 (5.44 SD)	22.0 (5.20 SD)	22.7 (5.64 SD)
Difference	2.26 (3.51 SD)	1.73 (3.74 SD)	2.75 (3.21 SD)
RM			
Asklepios	0.477 (0.0872 SD)	0.468 (0.0858 SD)	0.485 (0.0876 SD)
ARCSolver	0.529 (0.103 SD)	0.495 (0.0946 SD)	0.561 (0.0999 SD)
Difference	0.0526 (0.0830 SD)	0.0275 (0.0771 SD)	0.0755 (0.0815 SD)
PWV, m/s			
Asklepios	6.56 (1.31 SD)	6.57 (1.29 SD)	6.55 (1.34 SD)
ARCSolver	7.28 (0.931 SD)	7.51 (0.869 SD)	7.06 (0.935 SD)
Difference	0.717 (1.25 SD)	0.945 (1.26 SD)	0.510 (1.20 SD)
Zc, mmHg*s/ml			
Asklepios	0.109 (0.0377 SD)	0.101 (0.0341 SD)	0.116 (0.0394 SD)
ARCSolver	0.0986 (0.0248 SD)	0.0937 (0.0205 SD)	0.103 (0.0275 SD)
Difference	-0.0102 (0.0330 SD)	-0.00764 (0.0296 SD)	-0.0126 (0.0356 SD)
SVR, mmHg*s/ml			
Asklepios	1.28 (0.310 SD)	1.19 (0.271 SD)	1.36 (0.320 SD)
ARCSolver	1.21 (0.232 SD)	1.10 (0.165 SD)	1.31 (0.237 SD)
Difference	-0.0690 (0.281 SD)	-0.0898 (0.253 SD)	-0.0499 (0.304 SD)
SV, ml			
Asklepios	77.3 (18.0 SD)	85.9 (18.2 SD)	69.4 (13.7 SD)
ARCSolver	79.4 (14.3 SD)	89.7 (11.7 SD)	69.9 (8.93 SD)
Difference	2.09 (17.1 SD)	3.84 (18.7 SD)	0.495 (15.3 SD)

Difference, ARCSolver-Asklepios

Pf (Pb), amplitude of the forward (backward) traveling pressure wave

RM, reflection magnitude

PWV, pulse wave velocity

Zc, characteristic impedance

SVR, systemic vascular resistance

SV stroke volume. Results are given as mean (SD).

**Fig 4:**
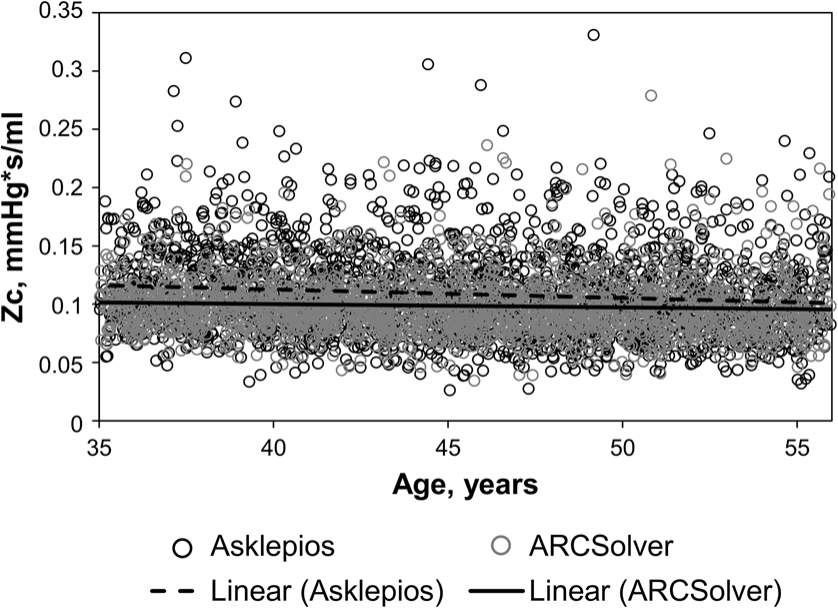
Characteristic impedance Zc over age.

Mean Zc estimated by the model was 0.0986 (0.0248 SD) mmHg*s/ml compared to 0.109 (0.0377 SD) derived from echocardiography with a mean difference of -0.0102 (0.0330 SD) and a correlation coefficient of R = 0.51. Similar effects for both approaches could be observed for the correlation to age as they were slightly negative (R = -0.07 for ARCSolver, R = -0.12 for Asklepios) as shown in Fig 4. In addition, the negative association was stronger in the male subgroup for both methods (men: R = -0.14 vs. R = -0.16; women: R = -0.02 vs. R = -0.09, ARCSolver vs. Asklepios). Table 2 shows the determinants of characteristic impedance for both approaches as well as for their difference.

**Table 2:** Analysis of determinants for characteristic impedance for both methods by a linear regression model (stepwise, enter if P<0.05, remove if P>0.1).

Variables	Coefficient	Standard Error	P Value	R partial
**Model for Asklepios Zc, adjusted R** ^**2**^ **= 0.2093**				
Gender	0.00691	0.00206	0.0008	0.07
Age, years	-0.00121	0.000128	<0.0001	-0.20
Height, cm	-0.000358	0.000116	0.0021	-0.07
BMI, kg/m^2	-0.00116	0.000179	<0.0001	-0.14
HR, bpm	-	-	-	-
PP carotid, mmHg	0.00129	0.0000638	<0.0001	0.39
**Model for ARCSolver Zc, adjusted R** ^**2**^ **= 0.3508**				
Gender	0.0109	0.00121	<0.0001	0.19
Age, years	-0.000738	0.0000756	<0.0001	-0.20
Height, cm	0.000214	0.0000691	0.0020	0.07
BMI, kg/m^2	-	-	-	-
HR, bpm	-	-	-	-
PP carotid, mmHg	0.00125	0.0000381	<0.0001	0.57
**Model for ARCSolver Zc–Asklepios Zc, adjusted R** ^**2**^ **= 0.0360**				
Gender	-	-	-	-
Age, years	0.000435	0.000120	0.0003	0.08
Height, cm	0.000417	0.0000784	<0.0001	0.11
BMI, kg/m^2	0.00104	0.000171	<0.0001	0.13
HR, bpm	-	-	-	-
PP carotid, mmHg	-	-	-	-

PP, pulse pressure

BMI, body mass index

HR, heart rate; gender, male = 1, female = 2.

Estimated pulse wave velocity within the cohort was 7.28 (0.93 SD) m/s and carotid to femoral PWV was 6.56 (1.31 SD) m/s with a mean difference of 0.717 (1.25 SD) m/s and R = 0.42. Scatter and Bland-Altman plot analysis (Fig 2C and 2F) shows only a slight although significant trend. Beyond a gender-specific offset, both methods show a similar behavior with regard to age as illustrated in Fig 3C.

Mean stroke volume is 79.4 (14.3 SD) ml for the mathematical model and 77.3 (18.0 SD) ml measured by echocardiography. Derived systemic vascular resistance (SVR) is therefore 1.21 (0.232 SD) mmHg*s/ml for modeled blood flow and 1.28 (0.310 SD) mmHg*s/ml for measured blood flow, respectively.

## Discussion

The aim of this work was the comparison between measured surrogates of arterial stiffness and wave reflections and estimated ones derived from a model based approach in the ASKLEPIOS cohort. As indicated by previous work, calculation of wave separation parameters (WSA) seems to be very robust due to their independence of absolute values with regard to Zc [8]. The WSA parameters showed a good agreement over the whole range of age and gender. Mean difference as well as standard deviation of both biomarkers (Pf and Pb) showed only slight trends for the residuals. The modest systematic offset in Pb may be due to the fact that the ARCSolver is designed to work with aortic waveforms which show a less steep upstroke in early systole in contrast to the carotid waveforms used in this study. This offset also caused a systematic overestimation of the reflection magnitude RM by the ARCSolver method compared to the Doppler flow. Thus, a potential correction of Pb will also affect RM positively and further investigations are therefore indicated. Nevertheless, the results already strengthen data on outcome published earlier for model-based methods [10–12].

While WSA parameters are supposed to serve as surrogates of pulsatile hemodynamics, cardiac output and systemic vascular resistance are seen to represent the ‘steady’ cardiovascular properties due to their relation to mean arterial pressure. The systemic vascular resistance shows similar trends over age for both methods. In more detail, the increase in SVR with age is more pronounced in women than in men for both methods. This increase over age is slightly more pronounced for the ARCSolver method. SVR is directly linked to stroke volume via mean arterial pressure and heart rate and therefore stroke volume shows a similar behavior in a mirrored way, see Fig 3D. Nevertheless, absolute values of stroke volume remain a challenge for currently available noninvasive measurement techniques.

Characteristic impedance represents the influence of the arterial wall and subsequently the link between pressure and flow. Zc is linked to arterial stiffness, vascular load and anthropometric measures. The direct comparison shows again acceptable agreement. The analysis of determinants for Zc showed similar contribution of carotid pulse pressure, sex and age for both methods but, in contrast to the Doppler based method, no significant influence of body mass index on the model based approach. The influence of height on Zc in both models is only modest and should be interpreted with caution, because the effects of body size are already partly covered by the gender variable. Similar effects were found in a recent study on untreated hypertensive subjects [17], where ARCSolver Zc was not related to BMI or body surface area. However Zc was significantly correlated with relative wall thickness and the left ventricular mass index. Furthermore, a slightly negative correlation with age was observed for Zc for both methods, which was more pronounced in male subjects. Such behavior has also been observed in other cohorts [28,29]. This seems to be a paradox phenomenon at a first glance but may be explained by a potential increase of aortic diameter with age in combination with a more pronounced stiffening of elastic arteries in women than in men in this age range [30–32].

Carotid to femoral pulse wave velocity is the actual non-invasive gold standard for the estimation of aortic pulse wave velocity (PWV) [7]. In a recently published meta-analysis PWV could demonstrate its additional predictive value beyond established risk scores for several target groups [6]. To spread its useful application, simplified acquisition approaches may help. The Windkessel based aortic PWV estimation method has already been compared in other cohorts with cfPWV and invasive aortic measurements [22, 23, 33,34]. Observed results were similar with those in this community and rating according to the ARTERY Society recommendations [35] turned out to be equal. In these recommendations, a mean difference < 1m/sec (with a SD of < 1.5 m/sec)–as observed in our study—between the gold standard and the comparator is classified as acceptable. However, further work may be beneficial to verify this agreement over the whole age range. The narrow age window might also explain the moderate correlation between cfPWV and ARCSolver PWV observed in this study, as age is the most important determinant of PWV. In a recent study, ARCSolver PWV from aortic pressure curves was closely related to cfPWV and especially invasive PWV over a wide age range [33]. Furthermore, prospective longitudinal outcome data of a chronic kidney disease stage 2–4 population was published supporting the predictive value associated with single point estimation of PWV by the ARCSolver method [25]. Upcoming assessment of adverse cardiovascular events in this cohort will help to provide further prospective evidence. Fig 3 reveals that cf-PWV in the Asklepios cohort develops similarly over age for men and women, while for ARCSolver PWV higher values for men but a more rapid increase for women can be seen. It remains unclear which progressions reflect true aortic PWV. Vermeersch et al. extensively investigated local and global stiffness behavior in the Asklepios cohort [32]. They found a steeper increase for women compared to men over age for local carotid PWV and higher values in local femoral PWV in men over all age groups. In several studies looking at pulse wave velocity development over age for both sexes (carotid-femoral as well as brachial-ankle PWV), the effect of a lower PWV in women at younger ages but a steeper increase leading to similar or higher PWV in older ages can be seen. These progressions are differently pronounced, depending on the specific study and cohort [36–39]. They show sometimes small but sometimes also significant differences.

### Limitations

In this study carotid pulse waves were applied to the ARCSolver algorithms instead of central aortic pulse waves for the first time. Therefore, slight adaptions in the signal processing chain were necessary although no modifications in the model as well as the algorithms themselves were performed. Nevertheless, the modifications possibly influence the current findings. Confirmation in other cohorts may be seen as useful. Furthermore, the study population consisted of middle-aged, healthy subjects only, thus results may not be generalizable to other cohorts. Because of the narrow age-range, this may be especially true for PWV.

## Conclusion

Overall, we observed acceptable agreement for all studied parameters. The results therefore indicate that the use of model-based surrogates in a healthy middle aged cohort is feasible and deserves further attention. Furthermore, this comparison implicitly also allowed a cross-validation of a potential operator dependent bias with regard to age and sex on cohort level for which no evidence could be found. For both methods, the parameters of arterial function compared here showed their predictive power already in prospective trials and independent cohorts. The presented results show consistency and strengthen previous findings.

## Supporting Information

S1 File Supporting Information. (DOCX)
