# Supplementary material for: Assessment of Model Based (Input) Impedance, Pulse Wave Velocity, and Wave Reflection in the Asklepios Cohort
Source: PLoS One. 2015 Oct 29;10(10):e0141656. doi: 10.1371/journal.pone.0141656 (PMC4626380; doi:10.1371/journal.pone.0141656)
Supplement: S1 File — (DOCX) [file pone.0141656.s001.docx]

**supporting information**

Assessment of model based (input) impedance, pulse wave velocity, and wave reflection in the ASKLEPIOS cohort

Hametner Bernhard^1,¶^, Parragh Stephanie^1,2^, Mayer Christopher^1^, Weber Thomas^3^, Van Bortel Luc^4^, De Buyzere Marc^4^, Segers Patrick^5^, Rietzschel Ernst^6^, Wassertheurer Siegfried^1,2,¶^

^1^ Health & Environment Department, AIT Austrian Institute of Technology, Vienna, Austria

^2^ Department of Analysis & Scientific Computing, Vienna University of Technology, Vienna, Austria

^3^ Cardiology Department, Klinikum Wels-Grieskirchen, Wels, Austria

^4^ Department of Pharmacology, Ghent University, Ghent, Belgium

^5^ Institute of Biomedical Technology, iMinds Medical IT, Ghent University, Ghent, Belgium

^6^ Department of Internal Medicine, Ghent University, Ghent, Belgium

* Corresponding author

E-mail: [Siegfried.Wassertheurer@ait.ac.at](mailto:Siegfried.Wassertheurer@ait.ac.at) (SW)

^¶^ These authors contributed equally to this work.

The Asklepios study comprises 2524 (1301 women) participants and their baseline characteristics were published before [1]. In the present study only participants with complete data were included, which reduced the number to 2292. Also, pressure curves with a delayed onset (57) and pulse wave velocities higher than 13.5 m/s (8) as well as one subject with a systolic blood pressure equal to 250 mmHg were excluded from the present analysis. This resulted in a total sample size of 2226 (1163 women). The basic clinical data is given in Table A.

To analyze the behavior of the hemodynamic parameters with respect to age and sex, the study population was divided into 4 age groups Q1-Q4 following [2]. The detailed results of this subgroup analysis are given in Table B.

Correlations between the hemodynamic parameters obtained with the ARCSolver and the original Asklepios methods are given in Table C.

**Supplementary References**

[1] Rietzschel ER, De Buyzere ML, Bekaert S, Segers P, De Bacquer D, Cooman L, Van Damme P, Cassiman P, Langlois M, Van Oostveldt P, Verdonck PR, De Backer G, Gillebert TC. Rationale, design, methods and baseline characteristics of the Asklepios Study. Eur J Cardiovasc Prev Rehabil 2007; 14:179–191.

[2] Segers P, Rietzschel ER, De Buyzere ML, Vermeersch SJ, De Bacquer D, Van Bortel LM, De Backer G, Gillebert TC, Verdonck PR, on behalf of the Asklepios investigators. Noninvasive (Input) Impedance, Pulse Wave Velocity, and Wave Reflection in Healthy Middle-Aged Men and Women. Hypertension 2007; 49:1248–1255.

| **Table A. Basic clinical data for the total population and for men and women separately.** | | | |
| --- | --- | --- | --- |
| Parameter | total (2226) | men (1063) | women (1163) |
| Age, years | 45.3 (5.87 SD) | 45.3 (5.85 SD) | 45.3 (5.89 SD) |
| Height, cm | 169 (8.88 SD) | 176 (6.49 SD) | 163 (6.1 SD) |
| Weight, kg | 73.5 (14.3 SD) | 81.4 (12.1 SD) | 66.3 (12.1 SD) |
| BMI, kg/m^2 | 25.6 (4.09 SD) | 26.3 (3.55 SD) | 24.9 (4.42 SD) |
| HR, bpm | 64.8 (9.62 SD) | 63.0 (10.1 SD) | 66.5 (8.82 SD) |
| SBP carotid, mmHg | 131 (16.4 SD) | 131 (14.7 SD) | 130 (17.8 SD) |
| DBP carotid, mmHg | 77.2 (10.8 SD) | 78.2 (10.6 SD) | 76.3 (10.9 SD) |
| MBP carotid, mmHg | 101 (12.1 SD) | 101 (11.5 SD) | 99.9 (12.6 SD) |
| PP carotid, mmHg | 53.4 (11.4 SD) | 52.9 (10.1 SD) | 53.8 (12.4 SD) |
| BMI, body mass index; HR, heart rate; SBP, systolic blood pressure; DBP, diastolic blood pressure; MBP, mean blood pressure; PP, pulse pressure. Results are given as mean (SD). | | | |

| **Table B. Hemodynamic parameters in men and women per age group.** | | | | | | | | |
| --- | --- | --- | --- | --- | --- | --- | --- | --- |
|  | Q1  35-40 years  277/300 (m/w) | | Q2  41-45 years  274/303 (m/w) | | Q3  46-50 years  265/281 (m/w) | | Q4  51-56 years  247/279 (m/w) | |
| Parameter | Asklepios | ARCSolver | Asklepios | ARCSolver | Asklepios | ARCSolver | Asklepios | ARCSolver |
| **Pf, mmHg** | | | | | | | | |
| **men** | 46.5 (9.90) | 48.4 (9.92) | 43.1 (8.94) | 44.8 (9.32) | 42.3 (9.29) | 43.3 (9.25) | 43.4 (9.36) | 43.8 (9.34) |
| **women** | 40.8 (8.17) | 41.0 (7.92) | 40.7 (8.33) | 40.4 (8.41) | 41.7 (9.40) | 41.0 (9.34) | 42.7 (9.66) | 41.3 (9.69) |
| **Pb, mmHg** | | | | | | | | |
| **men** | 20.5 (5.43) | 21.7 (5.23) | 20.1 (4.64) | 21.8 (4.85) | 20.2 (5.34) | 21.6 (4.85) | 20.6 (5.01) | 23.1 (5.74) |
| **women** | 18.6 (4.93) | 20.9 (4.66) | 19.4 (5.04) | 21.7 (5.21) | 20.3 (5.13) | 23.3 (5.61) | 21.8 (5.68) | 25.3 (6.06) |
| **RM** | | | | | | | | |
| **men** | 0.442 (0.0756) | 0.452 (0.0722) | 0.47 (0.0852) | 0.494 (0.091) | 0.481 (0.0889) | 0.506 (0.0993) | 0.479 (0.0879) | 0.533 (0.0966) |
| **women** | 0.459 (0.0779) | 0.512 (0.0838) | 0.479 (0.0864) | 0.543 (0.1) | 0.491 (0.0855) | 0.575 (0.0987) | 0.515 (0.0914) | 0.616 (0.0856) |
| **PWV, m/s** | | | | | | | | |
| **men** | 6.01 (0.974) | 6.95 (0.717) | 6.32 (1.04) | 7.23 (0.693) | 6.83 (1.20) | 7.67 (0.713) | 7.17 (1.56) | 8.28 (0.736) |
| **women** | 6.01 (1.140) | 6.30 (0.599) | 6.25 (1.10) | 6.73 (0.655) | 6.78 (1.26) | 7.32 (0.713) | 7.24 (1.50) | 7.99 (0.778) |
| **Zc, mmHg*s/ml** | | | | | | | | |
| **men** | 0.112 (0.0365) | 0.0997 (0.0207) | 0.0987 (0.031) | 0.0927 (0.0208) | 0.0983 (0.035) | 0.0914 (0.0196) | 0.0963 (0.0313) | 0.0906 (0.0199) |
| **women** | 0.121 (0.0388) | 0.104 (0.0245) | 0.117 (0.0403) | 0.102 (0.0242) | 0.113 (0.0401) | 0.103 (0.0288) | 0.111 (0.038) | 0.103 (0.0321) |
| **SVR, mmHg*s/ml** | | | | | | | | |
| **men** | 1.15 (0.240) | 1.03 (0.148) | 1.19 (0.281) | 1.09 (0.175) | 1.20 (0.268) | 1.12 (0.162) | 1.2 (0.292) | 1.15 (0.152) |
| **women** | 1.31 (0.296) | 1.21 (0.221) | 1.33 (0.306) | 1.27 (0.226) | 1.37 (0.318) | 1.34 (0.226) | 1.43 (0.347) | 1.43 (0.218) |
| **SV, ml** |  |  |  |  |  |  |  |  |
| **men** | 86.2 (18.3) | 93.2 (11.7) | 86.6 (18.7) | 91.3 (11.2) | 83.9 (17.5) | 86.5 (11.5) | 86.7 (18.5) | 87.5 (11.3) |
| **women** | 69.5 (13.4) | 73.3 (9.18) | 69.4 (13.6) | 70.5 (9.2) | 70 (13.8) | 69.2 (8.51) | 68.8 (14.1) | 66.3 (7.19) |
| Pf (Pb), amplitude of the forward (backward) traveling pressure wave; RM, reflection magnitude; PWV, pulse wave velocity; Zc, characteristic impedance; SVR, systemic vascular resistance; SV, stroke volume. Results are given as mean (SD). | | | | | | | | |

| **Table C. Correlations between the hemodynamic parameters obtained with the ARCSolver and the original Asklepios methods in the total population.** | | | | | | | | | | | | | | | |
| --- | --- | --- | --- | --- | --- | --- | --- | --- | --- | --- | --- | --- | --- | --- | --- |
|  |  | ARCSolver | | | | | | | Asklepios | | | | | | |
|  |  | Pf | Pb | RM | PWV | Zc | SVR | SV | Pf | Pb | RM | PWV | Zc | SVR | SV |
| ARCSolver | Pf |  | 0.63 | -0.34 | 0.62 | 0.75 | -0.18 | 0.42 | 0.90 | 0.60 | -0.23 | 0.03 | 0.49 | -0.06 | 0.22 |
|  | Pb | 0.63 |  | 0.50 | 0.58 | 0.50 | 0.41 | 0.10 | 0.66 | 0.79 | 0.31 | 0.22 | 0.30 | 0.28 | 0.10 |
|  | RM | -0.34 | 0.50 |  | 0.03 | -0.23 | 0.68 | -0.35 | -0.19 | 0.28 | 0.63 | 0.23 | -0.19 | 0.40 | -0.12 |
|  | PWV | 0.62 | 0.58 | 0.03 |  | 0.36 | 0.15 | 0.19 | 0.61 | 0.54 | 0.04 | 0.42 | 0.20 | 0.10 | 0.16 |
|  | Zc | 0.75 | 0.50 | -0.23 | 0.36 |  | 0.15 | -0.09 | 0.64 | 0.44 | -0.16 | -0.05 | 0.51 | 0.02 | 0.01 |
|  | SVR | -0.18 | 0.41 | 0.68 | 0.15 | 0.15 |  | -0.58 | -0.06 | 0.27 | 0.44 | 0.29 | -0.06 | 0.49 | -0.15 |
|  | SV | 0.42 | 0.10 | -0.35 | 0.19 | -0.09 | -0.58 |  | 0.30 | 0.13 | -0.17 | -0.16 | 0.02 | -0.17 | 0.46 |
| Asklepios | Pf | 0.90 | 0.66 | -0.19 | 0.61 | 0.64 | -0.06 | 0.30 |  | 0.70 | -0.20 | 0.07 | 0.66 | -0.04 | 0.20 |
|  | Pb | 0.60 | 0.79 | 0.28 | 0.54 | 0.44 | 0.27 | 0.13 | 0.70 |  | 0.54 | 0.20 | 0.42 | 0.19 | 0.12 |
|  | RM | -0.23 | 0.31 | 0.63 | 0.04 | -0.16 | 0.44 | -0.17 | -0.20 | 0.54 |  | 0.19 | -0.19 | 0.31 | -0.07 |
|  | PWV | 0.03 | 0.22 | 0.23 | 0.42 | -0.05 | 0.29 | -0.16 | 0.07 | 0.20 | 0.19 |  | -0.12 | 0.13 | 0.00 |
|  | Zc | 0.49 | 0.30 | -0.19 | 0.20 | 0.51 | -0.06 | 0.02 | 0.66 | 0.42 | -0.19 | -0.12 |  | 0.36 | -0.39 |
|  | SVR | -0.06 | 0.28 | 0.40 | 0.10 | 0.02 | 0.49 | -0.17 | -0.04 | 0.19 | 0.31 | 0.13 | 0.36 |  | -0.71 |
|  | SV | 0.22 | 0.10 | -0.12 | 0.16 | 0.01 | -0.15 | 0.46 | 0.20 | 0.12 | -0.07 | 0.00 | -0.39 | -0.71 |  |
| Correlations were assessed with Pearson’s correlation coefficient. Abbreviations as in Table B. | | | | | | | | | | | | | | | |
